# Tracking Acquired Antibiotic Resistance in Commensal Bacteria of Galápagos Land Iguanas: No Man, No Resistance

**DOI:** 10.1371/journal.pone.0008989

**Published:** 2010-02-01

**Authors:** Maria Cristina Thaller, Luciana Migliore, Cruz Marquez, Washington Tapia, Virna Cedeño, Gian Maria Rossolini, Gabriele Gentile

**Affiliations:** 1 Dipartimento di Biologia, University Tor Vergata, Roma, Italy; 2 Galápagos National Park Service, Puerto Ayora, Galápagos Islands, Ecuador; 3 Galápagos Genetics, Epidemiology and Pathology Laboratory “F. Valverde”, Galápagos National Park, Puerto Ayora, Galápagos Islands, Ecuador; 4 Concepto Azul, Guayaquil, Ecuador; 5 Dipartimento di Biologia molecolare, University of Sienna, Sienna, Italy; BMSI-A*STAR, Singapore

## Abstract

**Background:**

Antibiotic resistance, evolving and spreading among bacterial pathogens, poses a serious threat to human health. Antibiotic use for clinical, veterinary and agricultural practices provides the major selective pressure for emergence and persistence of acquired resistance determinants. However, resistance has also been found in the absence of antibiotic exposure, such as in bacteria from wildlife, raising a question about the mechanisms of emergence and persistence of resistant strains under similar conditions, and the implications for resistance control strategies. Since previous studies yielded some contrasting results, possibly due to differences in the ecological landscapes of the studied wildlife, we further investigated this issue in wildlife from a remote setting of the Galapagos archipelago.

**Methodology/Principal Findings:**

Screening for acquired antibiotic resistance was carried out in commensal enterobacteria from *Conolophus pallidus*, the terrestrial iguana of Isla Santa Fe, where: i) the abiotic conditions ensure to microbes good survival possibilities in the environment; ii) the animal density and their habits favour microbial circulation between individuals; and iii) there is no history of antibiotic exposure and the impact of humans and introduced animal species is minimal except for restricted areas. Results revealed that acquired antibiotic resistance traits were exceedingly rare among bacteria, occurring only as non-dominant strains from an area of minor human impact.

**Conclusions/Significance:**

Where both the exposure to antibiotics and the anthropic pressure are minimal, acquired antibiotic resistance traits are not normally found in bacteria from wildlife, even if the ecological landscape is highly favourable to bacterial circulation among animals. Monitoring antibiotic resistance in wildlife from remote areas could also be a useful tool to evaluate the impact of anthropic pressure.

## Introduction

The emergence and evolution of antibiotic resistance among bacterial pathogens has a global impact on public health. Antibiotic use in clinical, veterinary and agricultural practices has been the major selective force for emergence and spreading of resistant strains and resistance genes, since the 1950s [1]. However, the dynamics of dissemination and ecology of acquired antibiotic resistance (mediated by resistance determinants that are not present normally in bacteria) are very complex [2] and difficult to elucidate. In recent years, interesting insights into this field have been provided by studies on the presence of acquired resistance traits in the commensal microbiota of various wildlife animals [3], mostly mammals and birds from either remote or promiscuous settings. These studies, however, produced some conflicting results which stimulated discussions about the expedience of a strict control policy on antibiotics release for resistance control. Discussion mainly arose after the studies of Gilliver et al [4], who observed a notable prevalence of acquired antibiotic resistance traits in faecal bacteria from wild rodents living in woodland sites in northwest England where no antibiotic release was applied, and those of Österblad et al [5] who found an almost complete absence of resistance in faecal enterobacteria from wild rodents and ungulates living in remote areas of Finland. Based on the respective findings, the former study questioned the efficacy of restrictions in antibiotic use for resistance control, while the latter concluded that resistance reflects the use of antibiotics and resistance control would benefit from antibiotic restriction.

We hypothesize that the conflict could only be apparent and arise from differences between the ecological landscapes of the studied wildlife [4,5]. “Wildlife” includes all non-domesticated organisms, living in the most different ecological conditions, with dissimilar feeding and social behaviours. Thus, the meaning of collected data needs to be carefully scrutinized by applying a stringent systemic approach which accounts for the ecological context of the investigated organisms.

Therefore, this study was aimed at looking for the presence of acquired antibiotic resistance traits in commensal bacteria from wildlife living in a setting where such an approach could be applied. For this purpose, we have chosen the land iguana *Conolophus pallidus* living in Santa Fe, a small island in the Galápagos Archipelago, as the model of a remote setting where: i) abiotic factors are uniform and provide with good survival possibilities for microbes in the environment; ii) the studied wildlife is one of the most abundant terrestrial species, and its density and habits favour microbial circulation between individuals; iii) there is no history of antibiotic exposure, and the resident animal species are kept apart from man and introduced species, except for some restricted areas which may experience higher levels of human impact.

Although some natural antibiotic resistances were present (*e.g.,* beta-lactamases in some enterobacteria), acquired antibiotic resistance traits were found to be virtually absent in bacteria from the studied wildlife. When exceptionally present, they occurred in one of the areas with some human impact, being likely related to man.

## Results

**Figure 1:**
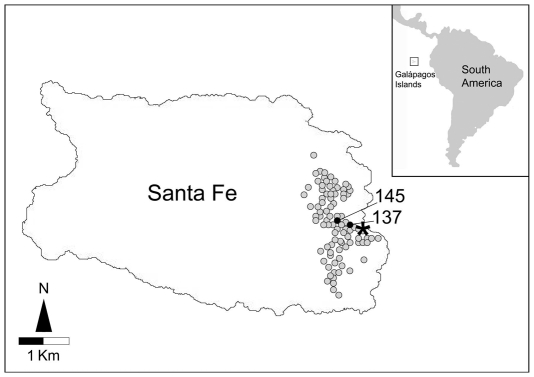
Santa Fe Island in the Galápagos Archipelago. Gray dots indicate the location of individuals sampled (coordinates datum WGS84, as recorded by a Garmin 12CX handheld GPS). Black dots indicate the individuals where acquired resistance was found. The asterisk marks the site occasionally visited by fishermen, scientists and TV-teams.

Cloacal swabs were taken from 96 adult iguanas, which represented approximately 10% of the total island population. The sampling area was designed to be located apart from the tourist path present in the island, but to also include a site accessed by local fishermen and where scientific expeditions or cine-TV troupes can settle their temporary camps (Fig. 1).

A confluent bacterial growth on MacConkey agar was obtained from all studied animals, revealing the constant presence of non-fastidious facultative or aerobic gram-negatives resistant to bile salts. The dominant component was always contributed by one or more species of the family *Enterobacteriaceae*. In most animals (88 of 96; 91.7%) a single dominant enterobacterial species was found, most often represented by *Escherichia coli* (n = 68), but also by *Citrobacter* spp. (n = 13) or *Klebsiella* spp. (n = 7). In the remaining animals (8 of 96; 8.3%) a shared dominance of two of the above species was observed.

Analysis of antibiotic susceptibility of the whole gut microbiota grown on McConkey agar to nine antibiotics (ampicillin, tetracycline, chloramphenicol, streptomycin, kanamycin, gentamicin, amikacin, nalidix acid, trimethoprim/sulphamethoxazole) yielded susceptibility profiles that were always consistent with the wild-type susceptibility profiles of the dominant (or co-dominant) enterobacterial species isolated from the same animal. This finding indicated that acquired resistance traits were apparently lacking in the dominant enterobacterial microbiota carried by the studied animals. Non-dominant resistant bacteria (*i.e.,* growing as single colonies within the inhibition zones of the antibiotic disks) were detected in several animals, mostly with ampicillin (*Citrobacter*, n = 27; *Enterobacter*, n = 15; *Klebsiella*, n = 12; *Serratia*, n = 11) and, less frequently, with other drugs including tetracycline (*Providencia*, n = 4), nalidixic acid (*E. coli*, n = 1), and gentamicin (*E. coli*, n = 1). All of the non-dominant resistant enterobacterial isolates belonged to a species that is naturally resistant to the corresponding antibiotic, except for the two *E. coli* isolates. The presence of non-dominant resistant isolates, therefore, indicated the occurrence of that species as a minor component of the gut microbiota and not of acquired resistance traits, except for the two non-dominant *E. coli* isolates resistant to nalidixic acid or gentamicin, which evidently carried acquired resistance traits.

These two *E. coli* isolates, from animals #137 and #145, named MCT-137 and MCT-145, were subjected to further characterization. MCT-137 was found to be resistant to nalidixic acid and tetracycline, and to carry a plasmid-mediated *tet*(B) gene, which could account for the acquired tetracycline-resistant phenotype and is a common acquired tetracycline resistance determinant in *E. coli* isolates of human origin [6]. MCT-145 was found to be resistant to gentamicin, streptomycin, kanamycin, chloramphenicol, tetracycline, ampicillin, nalidixic acid and trimethoprim/sulphamethoxazole, and to carry several plasmid-mediated resistance genes, including *aadB, aacA4, cat1*, *tet*(A), bla_TEM_ and *sul1*, , which could account for the acquired resistance phenotype to most of the above drugs and are common acquired resistance determinants in *E. coli* isolates of human origin [6]. MCT-145 also carried a class 1 integrase gene, while yielded a negative result for other common acquired resistance determinants including *aphaA*, *qnrA*, *qnrB*, and *qnrS*, *dfr8*, *tet*(B) and *sul2*. Interestingly, both MCT-137 and MCT-145 belonged in phylogroup B2 [7], which is fairly common among human isolates [8], [9], being different from the dominant antibiotic-susceptible *E. coli* isolates from the same animals, both of which belonged in phylogroup D.

## Discussion

Acquired antibiotic resistance traits were virtually absent in bacteria from the studied wildlife. The only exception was represented by two *E. coli* isolates, whose characteristics strongly suggest their exogenous, human origin, that were found in two different animals. In fact: i) their animal sources were both captured in the proximity of the site accessed by fishermen and where scientific expeditions and cine-TV troupes occasionally settle their camps; ii) the acquired resistance genes were the same as those commonly detected in resistant isolates from humans; iii) both isolates were detected at very low density within a background of dominant antibiotic-susceptible *E coli* of a different phylogroup.

Our results, therefore, are in overall agreement with the previous observations by Österblad et al [5] reporting a substantial lack of acquired antibiotic resistance in faecal enterobacteria from wildlife living in remote areas of Finland. The latter findings, however, were most likely favoured by the constrains of environmental factors (*e.g.,* long and cold winter, low population density, limited areas of overlapping with other animal species) that limited the circulation of enterobacteria between individuals and minimized the potential contamination by resistant strains from areas where humans and domesticated animals are living. Indeed, differences in these factors might have contributed to the different results reported by Gilliver et al [4], who found acquired resistance traits in faecal bacteria from the same wild rodent species, but living in woodland sites in England. The importance of the environmental factors is further outlined by the recent results of Sjölund et al [10], reporting drug-resistant *E. coli* from 8% of Arctic birds. These results seem to clash with those of the Finnish study, although obtained in an apparently similar environment. A deeper analysis points to the coexistence and crowding of different species, also endowed of migratory behaviour, as the factors that probably counterbalanced the obviously limiting abiotic ones in the Arctic environment.

In this regard, the Santa Fe Island is a unique model system, different from those previously studied. As all the islands of the Galápagos Archipelago it has never been connected to the mainland or inhabited by humans. The presence of *C. pallidus*, one of the three species of land iguanas endemic to the Galápagos Archipelago, that only occurs in Santa Fe and is listed as vulnerable in the Red List of the International Union for Conservation of Nature (IUCN) [11], justifies the strong protection of this setting from external influences. Tourist access is limited to over day excursions, following a restricted trail, and there is only a restricted site that can be accessed by local fishermen and by scientific expeditions or cine-TV troupes. On the other hand, in Santa Fe, the environmental survival and spreading of microbial strains are favoured by non-limiting abiotic factors (*e.g.,* mild temperatures, constant photoperiod and high humidity) and by animal density and their habits. In fact, similar to other iguanas, *C. pallidus* juveniles form their intestinal microbiota by coprophagy. Hence, bacteria colonizing the gut can easily spread within the reptile community and, if an introduced resistant strain should not need the presence of antibiotics to become widespread, in Santa Fe it would find the optimal conditions for this to occur. In this scenario, the detection of two *E. coli* isolates with acquired resistance traits of likely human origin as non-dominant microbiota in a small minority of animals, reveals that even highly isolated ecosystems are susceptible to contamination by multiresistant strains. However, in the absence of a chronic antibiotic exposure sustaining resistance, these strains failed to disseminate despite the fact that environmental conditions and animal habits were highly favourable to inter-individual spread, and that contamination from humans to wildlife could recurrently occur at that site.

A possible limitation of the present study is represented by the number of animals investigated, which did not include the whole population; however, the sampling strategy and the overall ecological landscape discussed above make it likely that the observed findings may reflect the actual situation encountered in the island wildlife.

In conclusion, the lack of acquired resistance traits in the dominant enterobacterial populations from wildlife, in this strictly controlled but microbes-friendly context, mimics a pre-antibiotic era situation and suggests that limited human-driven contamination, in the absence of a chronic antibiotic exposure, is not sufficient for the diffusion of acquired antibiotic resistance in wildlife.

## Materials and Methods

Cloacal swabs were collected from adult iguanas over a 9-day period, in February 2004, and stored (3–5 days) in Amies charcoal transport medium until processing at the Laboratory of Epidemiology, Parasitology and Genetics “F. Valverde” of the Galápagos National Park (Puerto Ayora, Santa Cruz). The overall antibiotic susceptibility profile of the enteric microbiota was assessed by a direct plating method [12], applying antibiotic-containing disks (Oxoid) on MacConkey agar plates uniformly inoculated with the swabs. Dominant species were detected by diluting a loopful of bacterial growth, taken from a confluent area well apart from the antibiotic disks. One colony for each observed morphology was picked up, stored in Cystine Triptose Agar (Difco) and sent to the reference microbiology laboratory in Italy, where they were checked for purity and stored at −70°C in glycerol 20% until examined. Identification, at least the genus level [13], and the determination of complete antimicrobial susceptibility profiles were performed by means of conventional methods [14], [15]. The same procedures were applied to the non-dominant resistant isolates.
